# The emerging and evolving evidence supporting creatine as an ergogenic aid: history and applications

**DOI:** 10.1080/15502783.2026.2646627

**Published:** 2026-03-23

**Authors:** Chad Kerksick, Drew Gonzalez, Jeffery Stout, Scott Forbes, Darren Candow, Tim Ziegenfuss, Robert Marshall, René Schwesig, Richard Kreider

**Affiliations:** a Exercise and Performance Nutrition Laboratory, Lindenwood University, St. Charles, MO, USA; b Exercise & Sport Nutrition Lab, Department of Kinesiology and Sports Management, Texas A&M University, College Station, TX, USA; c Occupational, Performance, and Nutrition Lab, Department of Kinesiology, Sam Houston State University, Huntsville, TX, USA; d School of Kinesiology and Rehabilitation Sciences, University of Central Florida, Orlando, FL, USA; e Department of Physical Education Studies, Brandon University, Brandon, MB, Canada; f Faculty of Kinesiology and Health Studies, University of Regina, Regina, SK, Canada; g The Center for Applied Health Sciences, Canfield, OH, USA; h AFC Bournemouth, Department of Performance and Medicine, Bournemouth, United Kingdom; i Department of Orthopedic and Trauma Surgery, Martin-Luther-University Halle-Wittenberg, University Medicine, Halle, Germany

**Keywords:** Performance, recovery, safety, team sports, tactical athletes, endurance

## Abstract

**Background:**

Creatine is one of the most extensively studied ergogenic aids, with over three decades of research supporting its role in exercise performance, recovery, and health.

**Methods:**

This narrative review summarizes the historical development of creatine supplementation and evaluates evidence regarding its mechanisms, efficacy across active, athletic populations (e.g. strength, endurance, team-sport), and tactical (e.g. military, law enforcement) populations, and its safety profile.

**Results:**

The evidence suggests that creatine enhances phosphocreatine resynthesis and cellular energy availability, resulting in consistent improvements in high-intensity exercise performance, training adaptations, lean body mass, strength, and power. Additional findings indicate that creatine may attenuate exercise-induced muscle damage and inflammation, support recovery, and improve functional outcomes following strenuous activity. Emerging research suggests benefits for endurance and team-sport athletes through enhanced glycogen resynthesis, calcium handling, oxidative stress mitigation, and repeated-sprint performance. In tactical populations, creatine may support occupational readiness by improving strength, hydration status, thermoregulation, cognition, sleep quality, and recovery, with possible neuroprotective and cardiometabolic implications. Soccer-specific evidence demonstrates improvements in repeated-sprint ability and tolerance to high training loads, with preliminary data suggesting protective effects against neurotrauma and gut barrier disruption. Importantly, pooled analyses from hundreds of clinical trials report no greater incidence of adverse events compared with placebo, reinforcing creatine's established safety profile.

**Conclusion:**

Overall, the evidence suggests that creatine is a versatile supplement with strong evidence to enhance performance and recovery across diverse populations. Future research should prioritize individualized dosing strategies, long-term outcomes in underrepresented groups, and exploration of novel therapeutic applications in health and disease

## 1. Introduction

**Figure 1.**
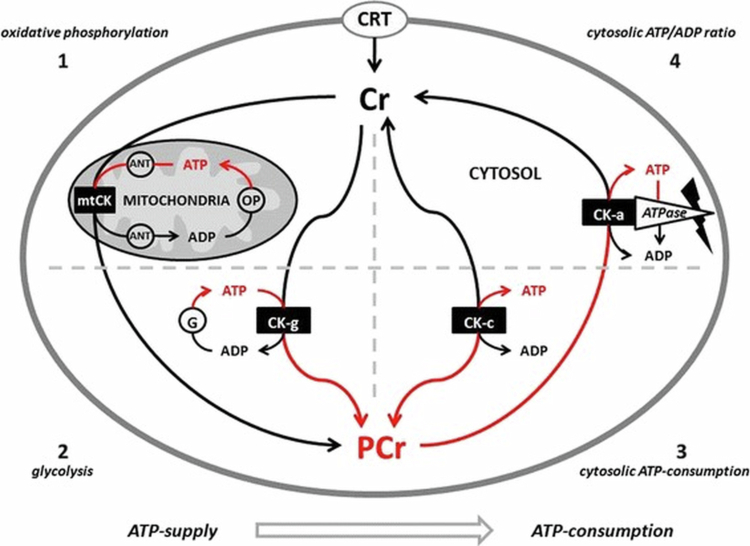
Proposed creatine kinase/phosphocreatine (CK/PCr) energy shuttle. CRT = creatine transporter; ANT = adenine nucleotide translocator; ATP = adenine triphosphate; ADP = adenine diphosphate; OP = oxidative phosphorylation; mtCK = mitochondrial creatine kinase; G = glycolysis; CK-g = creatine kinase associated with glycolytic enzymes; CK-c = cytosolic creatine kinase; CK-a = creatine kinase associated with subcellular sites of ATP utilisation; 1–4 sites of CK/ATP interaction. From Kreider et al. [1].

Creatine (*N*-(aminoiminomethyl)-*N*-methyl glycine) is a naturally occurring compound synthesised in the body from glycine, arginine, and methionine and can also be obtained from animal-based proteins or commercially available dietary supplements. Creatine is primarily stored in skeletal muscle (~95%) and plays a key role in regulation of adenosine triphosphate (ATP) supply during metabolically challenging tasks [1,2]. After ingestion and absorption, creatine is transported into the bloodstream and distributed to various storage sites, including the muscles, brain, and testes. Typically, a 70-kg male has ~90–160 mmol of total creatine per kilogram of skeletal muscle [3]. Increasing muscle creatine levels results in concomitant increases in intramuscular phosphocreatine (PCr), which is essential for rapidly restoring and maintaining ATP during high-intensity exercise through modulation of the creatine kinase/PCr energy shuttle [1] (Figure 1). In healthy people, about half of the daily creatine intake comes from endogenous synthesis, while the rest mainly comes from diet, especially meat and fish. While multiple versions of creatine supplements have been commercially produced, creatine monohydrate (CrM) has been studied the most. CrM contains 88% creatine by weight with creatine ions bound to water that readily separate during digestion. Studies have demonstrated that CrM supplementation raises free creatine, PCr, and total creatine levels in skeletal muscle by 20%–40% while observing excellent (i.e. >99%) bioavailability [2]. Furthermore, initial studies by Harris et al. [4] demonstrated robust increases in plasma creatine after one hour of ingestion, which were later confirmed by Persky et al. [5] and reviewed by Kreider et al. [2]. Individual variability in absorption has been observed and is largely attributed to variations in creatine transporter expression [2], which are secondary to genetic differences and dietary intake. Due to its impact on energy metabolism, much of CrM's initial interest centres upon its ability to function as an ergogenic aid in competitive and recreational athletes [1,6], with its widely reported ability to improve in high-intensity exercise capacity and heighten exercise training adaptations [1,6–11]. More recently, CrM applications have been extended to endurance and team sports, as well as tactical and occupational populations. This narrative review summarises current perspectives on CrM use across sports and populations, beginning with its historical development and biochemical roles, followed by performance outcomes and applications in endurance athletes, tactical populations, and soccer players, and concluding with a focused evaluation of safety and overall implications.

## 2. History of creatine in exercise and sport

Historically, creatine supplementation in sports has been closely linked to advancements in muscle biopsy techniques, which were developed in the 1960s by Jonas Bergström and Eric Hultman [12]. These early biopsy studies demonstrated the rapid depletion and recovery of PCr during and after intense exercise, highlighting the crucial role of creatine in energy metabolism [13–15]. However, initial human supplementation studies, particularly a 1975 pilot study led by Dr. Roger Harris [16], produced misleading results mainly due to methodological limitations and unusually high baseline muscle creatine levels in some subjects. Interest in creatine resurfaced in the late 1980s when Harris revisited creatine supplementation after providing creatine to a racing equine [12]. This led to the seminal 1992 study by Harris, Söderlund, and Hultman [17], which demonstrated significant increases (~20%) in total creatine and PCr concentrations following CrM supplementation. Subsequent foundational studies in the early to mid-1990s further confirmed the ergogenic effects of creatine. Greenhaff et al. [18,19] and Casey et al. [20] identified improvements in muscular strength, repeated sprint performance, power output, and fatigue resistance due to increased PCr availability, providing strong scientific support for creatine supplementation in athletic populations. These collective results underscore the importance of how early discoveries shaped methodological rigour in later research, positioning CrM as a scientifically backed nutritional strategy for improving athletic performance and health outcomes [1,6,7,21–27].

## 3. Creatine and performance metrics

Extensive research has demonstrated that CrM supplementation improves exercise performance outcomes [6]. In particular, supplementing with CrM for both short- and long-term durations can enhance high-intensity, short-duration physical performance (i.e. increase force production, velocity, and power output, improve recovery between sets, and increase work capacity), as well as promote long-term training adaptations [12,28]. The following sections provide an overview of how CrM can be leveraged in terms of its ergogenic effects.

### 3.1. Short-term creatine monohydrate supplementation

Short-term supplementation protocols with CrM typically include a loading phase (20 g/day or 0.3 g/kg of lean body mass for 7 days) followed by a maintenance phase (5 g or 0.075 g/kg of lean body mass). To date, much of the literature surrounding the loading dose and duration of CrM supplementation supports the augmentation of intramuscular creatine stores. Universal increases in PCr stores do not occur in response to these supplementation regimens leading to deeper discussion surrounding ‘responders’ vs. ‘non-responders’. In this respect, multiple factors such as baseline intramuscular creatine levels, age and diet, muscle fibre type and size function as key factors that can determine how an individual responds to supplementation [29]. Nonetheless, several studies report improvements in muscular strength and power in as little as five days [30–33]. In this respect, Cox et al. [34] reported improvements in repeated sprint and agility performance in elite female soccer players who consumed CrM (20 g/d for 6 days). A review of CrM’s immediate ergogenic effects combined with resistance training revealed an 8% increase in muscular strength compared to resistance training alone [35]. There is also some evidence to suggest that short-term CrM supplementation (i.e. 0.3 g/kg for 5 to 7 days) can improve fatigue resistance among athletes performing repeated exercise bouts [36,37]. For example, Birch et al. [38] demonstrated improved repeated cycling performance following five days of CrM (4 × 5 g/day), suggesting a higher sustained power output during repeated cycling sessions.

### 3.2. Chronic supplementation

While creatine's primary mechanism to enhance short-term performance is increased intramuscular PCr that improves high-energy phosphate buffering, the documented gains in strength and hypertrophy of skeletal muscle are secondary and arise from the ability to sustain higher training volumes and intensities over repeated training sessions [8,39]. Chronic CrM supplementation typically involves orally ingesting ~5 or 0.075 g/kg/day following 5–7 days of CrM loading. Notably, Hultman et al. [40] demonstrated that even without a loading phase, a 3 g dose for four weeks can maximally saturate intramuscular PCr stores. As demonstrated in numerous studies, sustained CrM use can enhance exercise training adaptations by facilitating greater training volume [8,41–45]. For example, male powerlifters saw improvements in bench press strength, endurance, and body mass after 26 days of CrM supplementation [43]. Volek et al. [46] demonstrated that 12 weeks of CrM supplementation, which involved taking 25 g/day for seven days of loading followed by 5 g/day for 11 weeks, among healthy resistance-trained men, led to increased body mass (6.3%) and fat-free mass (6.3%) compared to a placebo. Additionally, the researchers observed increases in the cross-sectional areas of type I (35%), IIA (36%), and IIAB (35%) muscle fibres, indicating CrM's potential to enhance training adaptations.

### 3.3. Creatine to accelerate recovery after muscle damage

#### 3.3.1. Mechanistic context

Exercise-induced muscle damage arises from primary structural strain (notably during eccentric actions) and a secondary inflammatory–oxidative response. Mechanical disruption at the myofibrillar and cytoskeletal levels precipitates transient strength loss and ultrastructural changes, followed by immune cell infiltration, cytokine release (e.g. interleukin-6 [IL-6], tumour necrosis factor-alpha [TNF-*α*]), and elevated reactive species that aid repair but can exacerbate injury if excessive [47]. Creatine may support recovery by bolstering ATP resynthesis capacity, influencing cellular hydration/osmotic signalling, and modulating redox-related pathways [6,48] – all of which can help preserve excitation-contraction coupling and membrane integrity during the vulnerable post-exercise window.

#### 3.3.2. Mixed findings from controlled trials

Early short-term (5 days) loading studies in untrained participants often reported no between-group differences in canonical damage markers (e.g. creatine kinase [CK], lactate dehydrogenase [LDH]) or symptoms, possibly because the magnitude and variability of the damaging bout masked modest treatment effects [6,49–53]. In contrast, protocols combining creatine with carbohydrate (CHO) around a damaging stimulus have shown functional benefits despite mixed biomarker responses. For example, Cooke et al. [49] reported faster strength recovery (1–4 days) and attenuated increases in damage markers (2–7 days after eccentric exercise) when CrM was co-ingested with CHO before and after eccentric exercise. Other trials have noted improvements in range of motion, limb girth/elasticity, and strength restoration, even when soreness or select urinary markers (e.g. titin) were unchanged [54]. Collectively, these data suggest creatine's most consistent early advantage is the restoration of function, with biomarker responses more variable across designs and cohorts.

#### 3.3.3. Synthesis, magnitude, and timing effects

Across 23 studies, meta-analytic evidence indicates that creatine can blunt rises in CK, LDH, IL-6, TNF-*α*, and indices of lipid peroxidation following strenuous exercise, while acknowledging that higher training volumes over time can elevate some markers as part of adaptation [55]. Practically, benefits appear largest in the immediate days post-exercise (i.e. during early functional recovery), with less consistent effects during later phases of training adaptation when remodelling predominates.

## 4. Special populations and considerations: endurance athletes

Creatine is a well-established ergogenic aid to enhance resistance training adaptations, including gains in lean body mass, strength, and power [1,6]. However, the impact of creatine supplementation on endurance performance is inconclusive and comparatively unexplored [56]. Mechanistically, several purported benefits of creatine supplementation may enhance endurance performance [56]. For example, creatine co-ingested with CHO may increase glycogen resynthesis, an important fuel for endurance exercise [57]. In addition, creatine supplementation increases intramuscular PCr and free creatine levels, which are important for rapid ATP resynthesis to support high-intensity bursts of muscle performance, such as sprinting and/or surges during a race [58]. Creatine supplementation may also help attenuate markers of inflammation [59] and oxidative stress following endurance exercise, thereby facilitating recovery [48,60]. Further, there is evidence that creatine helps shuttle ATP from the mitochondria to sites of utilisation (i.e. actin-myosin cross-bridge, sub-sarcolemma, sarcoplasmic reticulum, glycogen), which in theory could improve exercise capacity [61]. In an animal model, creatine supplementation maintained fast-twitch muscle fibre characteristics following chronic low-frequency stimulation, which mimics high-volume endurance training, without any detrimental effect on oxidative adaptations [62]. In contrast, creatine supplementation is often associated with a small increase in body mass (~0.86 kg) over time [63], which could negatively impact exercise economy and weight-bearing endurance performance [64]. As such, the purported performance effects appear to be a balance of the position effects vs. the gain the body mass, which varies between individuals (possibly due to baseline values and fibre type distribution), exercise modality (i.e. weight bearing, vs. non weight bearing), and the specific demands of the race (e.g. repeated sprints, change in terrain, hills, etc.).

### 4.1. Impact on VO_2_Max and endurance performance

#### 4.1.1. VO_2_Max and continuous exercise

Recently, two systematic reviews have examined the effects of creatine supplementation on endurance performance [26,65]. Gras et al. [26] found that creatine supplementation impaired absolute values of maximal oxygen consumption (VO_2_Max) (effect size −0.2: 95% CI −0.039 to −0.001, *p* = 0.049), but did not affect relative VO_2_Max, despite the typical increase in body mass associated with creatine supplementation [26,63]. Importantly, 80% of the 424 participants (~30 years of age) examined (82% male) were engaged in exercise training, which included both healthy and unhealthy clinical populations, and utilised studies that estimated VO_2_Max [26]. This heterogeneity may have influenced the overall conclusion, and the generalisability to endurance athletes is limited. To further address this, Fernández-Landa et al. [65] explored the effects of creatine supplementation on endurance performance in trained athletes. Both systematic reviews by Gras et al. [26] and Fernández-Landa et al. [65] found no benefit from creatine supplementation on exercise time to exhaustion. However, Gras et al. [26] did find improvements in ventilatory threshold in a sub-analysis of younger adults, which is an important indicator of race pace and endurance performance. To date, only one study has found a negative impact on running performance from creatine supplementation, which the authors suggested was related to an increase in body mass [26]. In contrast, others have assessed whether an increase in body mass affects non-weight-bearing exercises, such as cycling uphill (e.g. at an 8% incline), but have found no effect of creatine-mediated weight gain on exercise time to exhaustion [66].

#### 4.1.2. Sprints and supra-maximal exercise

Although creatine supplementation does not affect performance during lower-intensity continuous exercise, a growing body of evidence suggests that it can improve bursts of high-intensity exercise interspersed during an endurance event. For example, Tomcik et al. [66] conducted a 120-km time trial with trained cyclists, where participants alternated every 10 km between 1- and 4-km sprints. Results showed that creatine supplementation combined with CHO improved the final 1- and 4-km sprints, and the authors suggested that creatine may be a viable supplement to help with late-stage breakaways [66]. In support of these findings, Engelhardt et al. [67] found an 18% improvement in power output following creatine supplementation during high-intensity intervals (15-second sprints with 45-second rest repeated 10 times) when performed after 30 minutes of continuous exercise in triathletes. Furthermore, Anomasiri et al. [68] found that creatine supplementation improved the final finishing sprint (the last 50 m) in a 400-m swimming time trial. Graef et al. [69] investigated the effects of combining creatine with high-intensity interval training in active males and found improvements in time to exhaustion and ventilatory threshold with no changes in total work after supplementing with CrM for 5 days/week of 10 g/day for 30 days when compared to placebo. In contrast, Forbes et al. [70] replicated this study in females and did not find these improvements, which may highlight potential sex-based differences. These sex-based differences may be associated with females having higher baseline intramuscular creatine content prior to initiating supplementation compared to males do [71]. Syrotuik and Bell [29] demonstrated that “responders” to creatine had lower levels of baseline creatine content, as such females may be less responsive. Further research is urgently needed, since oestrogen can impact CK and the effects of creatine may be altered across the menstrual cycle [72,73]. To date, there is no direct evidence that has explored sex-based differences of CrM supplementation on endurance performance. Kendall et al. [74] found ergogenic effects on critical power in men who supplemented with CrM but not on anaerobic work capacity in high-intensity interval training. Furthermore, 3 g/day of CrM supplementation for 28 days improved exercise economy but not the respiratory exchange ratio (RER), blood lactate, or sprint performance at supramaximal speeds in endurance-trained males [75].

### 4.2. Practical implications

In summary, creatine is not a classical endurance enhancer for steady-state performance, but it shows promise in supporting high-intensity bursts within endurance sports and aiding recovery. In this respect, CrM has the potential to enhance aspects of endurance performance by increasing glycogen resynthesis when co-ingested with CHO and appears to help preserve the characteristics of fast-twitch muscle fibres during high-volume training. In addition, creatine supplementation increases intramuscular creatine levels, supporting high-intensity exercise capacity and reducing measures of inflammation and oxidative stress, which may facilitate recovery. Endurance athletes should weigh the small potential body mass gain against these mechanistic benefits based on individual responses.

## 5. Special populations and considerations: tactical athletes

### 5.1. Creatine and tactical athletes

**Figure 2.**
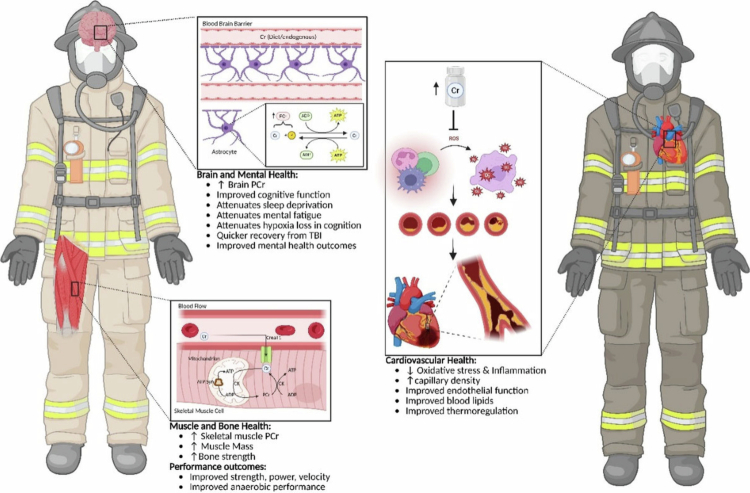
Creatine benefits for firefighters.

Tactical athletes face extreme conditions that can impair performance, accelerate aging, and elevate health risks. To address this, scientists and practitioners seek practical strategies to enhance health and performance, ensuring these personnel are occupationally ready [76,77]. CrM has gained attention for its ability to increase intramuscular creatine levels and to improve exercise performance and training adaptations [1]. In addition, emerging data demonstrate that CrM can improve sleep [78] and various aspects of the brain [22,79–84], as well as vascular [85–87], bone [23,88–91], and mental health. It is also worth noting that CrM has been suggested to play a role in injury prevention [92,93], thermoregulation [94–98], recovery [6,12,99], and may also provide prophylactic benefits following sustained or traumatic brain injury (TBI) [79,81,100–102]. Figure 1 outlines the multiple proposed applications of creatine for firefighters and other tactical athletes (Figure 2).

### 5.2. Performance benefits and safety of creatine monohydrate

While robust ergogenic evidence exists in athletes [1,6], occupational-specific data remain limited and somewhat mixed [24,25,103,104]. Existing research often features small sample sizes, brief intervention periods, and varied outcome measures. The findings have been inconsistent, especially when evaluating complex occupational tasks. Although creatine supplementation seems physiologically plausible to improve occupational performance, more well-controlled studies involving firefighters, law enforcement personnel, and military members are needed.

To date, only a few studies have assessed the impact of creatine supplementation among tactical and occupational athletes. First, de Silveira et al. [24] found that after 12 weeks of CrM and glutamine supplementation (i.e. both at a dose of 0.3 g/kg/d for 7 days followed by 0.03 g/kg/day for a 11-week maintenance phase) did not confer an ergogenic benefit on physical performance among 32 male military police officers. In addition, Warber et al. [104] also demonstrated that CrM supplementation (i.e. 24 g of CrM within a sports bar, 1 bar/day) for five days did not improve performance on a military obstacle course. On the contrary, Bennett et al. [103] demonstrated that CrM supplementation (i.e. 20 g/day for 6 days followed by 6 g/day of 4 weeks) led to an increase in the total number of pull-ups performed. Elstad et al. [25] conducted the only study using a first-responder cohort. In this study, they assessed if CrM supplementation (5 g/d) could improve occupational performance on a simulated battery of firefighting tasks while co-ingestion 25 g of whey protein isolate and 25 g of CHO each day for three weeks. The research team found that the CrM group improved their time to completion of two firefighting tasks: the victim dummy drag (1.78 ± 0.57 s) and forcible entry (2.66 ± 0.97 s). Currently, evidence regarding the ergogenic benefits of CrM for occupation-specific performance is mixed and more research is needed [76].

### 5.3. Other benefits of creatine monohydrate relevant to the tactical athlete

#### 5.3.1. Body composition

Body composition is an important determinant of health and occupational performance in tactical populations, with increasing rates of overweight and obesity reported in certain groups [77,105]. Excess adiposity and reduced lean mass may negatively influence physical readiness [77]. Preliminary data suggest that CrM, when combined with resistance training, benefits body composition [21,90,106,107]. A recent meta-analysis also reported a modest reduction in body fat percentage (−1.19%; *p* = 0.006) when CrM was combined with resistance training in young adults [108]. In addition, evidence indicates that CrM, when coupled with resistance training and walking, can improve bone health [88–91].

#### 5.3.2. Cardiovascular and antioxidant impacts

It is well-established that tactical and occupational athletes are susceptible to cardiometabolic and chronic disease due to the nature of their occupation (i.e. exposures to stressors) [77]. Emerging research has examined whether CrM may influence vascular function, lipid profiles, and markers of oxidative stress and inflammation [85–87]. For example, Clarke et al. [87] reported improved brachial artery flow-mediated dilation following 30 days of supplementation, while other studies have shown increases in limb blood flow. Other reports have shown favourable outcomes related to cardiovascular/cardiometabolic health. For instance, Arciero et al. [109] found that following a 5 × 4 g/d for a 5-d loading phase followed by 10 g/d maintenance phase in combination with resistance training among 30 healthy, untrained male subjects led to an increase (*p* < 0.05) in calf (30%) and forearm (38%) limb blood flow. Santos et al. [60] showed that runners taking 20 g/d for five days experienced attenuated changes in prostaglandin E2 (PGE2) (61%) and TNF-*α* (34%), while the placebo group experienced 6.6-fold and 2.3-fold increases for PGE2 and TNF-*α*, respectively. Accordingly, while preliminary findings suggest possible cardiometabolic relevance, additional controlled trials in occupational settings are warranted.

#### 5.3.3. Creatine supplementation and thermoregulation

Data suggest that CrM may benefit tactical athletes by improving thermoregulation [76], which is particularly important for firefighters. Thermal strain and dehydration are significant occupational concerns for firefighters and other tactical personnel operating in high-heat environments [110,111]. Creatine is supported by previous reports demonstrating that CrM can increase total body water and intracellular fluid volumes [94,97,112,113], which, in turn, are believed to help maintain plasma volume and improve heat dissipation and core body temperature. Supporting this concept, Easton et al. [114] reported that participants who followed a seven day CrM and glycerol supplementation protocol increased their total body water (CrM + Glycerol: 0.87 ± 0.21 L; CrM alone: 0.63 ± 0.33 L; glycerol + placebo: 0.50 ± 0.28 L), thus it seems reasonable to hypothesise that CrM may have a synergistic effect with glycerol in respect to fluid balance, but this needs to be directly examined among firefighters.

#### 5.3.4. Creatine and mental and brain health

Significant interest currently exists surrounding creatine’s potential to support brain bioenergetics, cognitive performance, and mental health outcomes. In this respect, studies are available that support creatine's ability to augment short-term memory, attention, and reasoning [22,82,115] along with attenuations in cognitive performance during periods of mental fatigue or sleep deprivation [84,116]. In terms of mental health, it is estimated that about 30% of the United States population is impacted by mental health disorders annually [82], and in tactical personnel, prevalence rates for post-traumatic stress disorder (PTSD) and depression are 4%–37% and 11%–40%, respectively [117]. Since creatine can cross the blood‒brain barrier (albeit at a slow rate), it may increase brain creatine levels and support brain bioenergetics [118], underscoring its mechanistic capacity to improve mood and mental health. For example, Roitman et al. [119] demonstrated reductions in depression and fatigue-related symptoms among those with treatment-resistant depression who were supplemented with CrM. Another area of interest centres upon the ability for CrM supplementation to aid in recovery from a traumatic brain injury (TBI) [120]. While much more research is needed, the current mechanistic link centres upon the costly bioenergetic nature of concussions and TBI, and that, if brain creatine content is increased secondary to CrM supplementation, the individual may be better able to handle this bioenergetic crisis. To date, the limited evidence in children with moderate to severe TBI indicates that CrM supplementation reduces symptoms of headache, dizziness, and fatigue compared with placebo [101,102]. Lastly, sleep, critical to health and performance, is particularly challenging to tactical and occupational athletes due to the widespread interruptions in their sleep environment [77]. Two recent studies have highlighted CrM's potential to support sleep outcomes [78,121]. For instance, Gordji-Nejad et al. [121] found that a single dose of CrM (0.35 g/kg) reversed metabolic alterations and fatigue-related cognitive impairments during a short-term period of sleep deprivation. Moreover, Aguiar Bonfim Cruz et al. [78] demonstrated that 5 g/d for six weeks of CrM supplementation in exercising women led to improved sleep (~1 hour more of sleep) on workout days. Overall, research indicates that creatine could affect cognitive function, mood, and sleep; however, evidence within tactical and occupational groups is still limited.

## 6. Special populations and considerations: creatine and soccer performance

### 6.1. Requirement profile and current developments in soccer

Soccer, in general, is a team sport characterised by athletic performance that combines strength and endurance, technical abilities, and a strong mindset. Soccer players must perform many intense, short anaerobic activities housed within a collective athletic effort powered by aerobic metabolism. There can be up to 200 intense actions during a match, with total match time rarely exceeding 90 minutes of net exposure [122,123]. Even during intense soccer-specific training, high-intensity actions with corresponding time in the highest levels of heart rate responses are common [10]. Only tactical or technical training takes place in the purely aerobic heart rate zones. This combination of intermittent anaerobic performance peaks with recurrent aerobic breaks highlights the importance of homoeostasis between creatine and PCr status for optimal soccer performance. Previous reports have indicated that intramuscular PCr levels can drop to about 60% of basal levels through competitive and training activities [122]. The demands of professional soccer continues to grow as the style of play is more intense, the number of games has increased, rest periods have shortened, and sometimes lengthy travel can disrupt sleep patterns and increase stress.

### 6.2. Creatine’s relationship to several performance effects

Creatine administration increases anabolic pathways and decreases catabolic pathways. Creatine supplementation increases strength by 20%–30% compared to a placebo, particularly when strength training is performed in isolation. As shown in Table 1, an average increase of 13% (placebo) and 24% (creatine) has been observed with benefits being realised for sports involving sprinting, jumping, throwing, or team sports with these activities.

**Table 1.** Creatine and isolated strength development based on various exercises using the 1RM test criterion.

Reference	n	Study duration (days)	Placebo (%)	Creatine (%)
Squats – performance Increases in strength (% 1RM)				
Vandenberghe et al. [45]	19	70	25	46
Volek et al. [39]	19	84	24	32
Pearson et al. [124]	16	70	0	11
Stone et al. [125]	20	35	8	12
Larson-Meyer et al. [126]	14	91	12	24
Bemben et al. [127]	17	63	5	9
Jowko et al. [128]	21	21	3	14
			**11**	21
Leg press – performance Increases in strength (% 1RM)				
Vandenberghe et al. [45]	19	70	25	43
Jowko et al. [128]	21	21	29	54
Peeters et al. [129]	35	42	10	12
Syrotuik et al. [44]	21	37	8	16
Arciero et al. [109]	30	28	16	42
Willoughby et al. [130]	16	84	29	54
			**20**	37
Bench press – performance Increases in strength (% 1RM)				
Vandenberghe et al. [45]	19	70	38	45
Volek et al. [39]	19	84	16	24
Pearson et al. [124]	16	70	0	3
Stone et al. [125]	20	35	4	10
Larson-Meyer et al. [126]	14	91	9	18
Bemben et al. [127]	17	63	0	5
Jowko et al. [128]	21	21	4	11
Peeters et al. [129]	35	42	1	10
Syrotuik et al. [44]	21	37	7	9
Arciero et al. [109]	30	28	9	18
Earnest et al. [131]	28	28	0	6
Noonan et al. [132]	25	56	0	6
Stout et al. [133]	24	56	5	7
Brenner et al. [134]	16	35	7	17
			**7**	13

Note: the numbers in bold in each section of the table are calculated averages of each group.

In particular, several studies have demonstrated an increase in activities directly translatable to on-field soccer performance, such as sprinting, jumping ability [135], and enhanced resistance to fatigue, independent of age and gender, in the pre-season period [136,137]. To this point, Mujika et al. [135] found improved sprint performance in creatine-supplemented soccer players, and Claudino et al. [136] observed enhanced lower-limb power during preseason training with CrM. These outcomes are particularly relevant as they directly highlight instances where creatine supplementation was shown benefit aspects of on-field performance. For further support, creatine supplementation in a comprehensive meta-analysis was shown to have a statistically significant effect on anaerobic metabolism and power (effect size: 1.23, [95% CI: 0.55 to 1.91]), particularly compared to athletic populations consuming a placebo while the same authors found no positive effects concerning aerobic capacity (effect size: −0.05, [95% CI: −0.38 to 0.28]) [138], a point that was discussed earlier in this paper.

### 6.3. Creatine as a neuroprotector or support agent for the gut and inflammation

**Figure 3.**
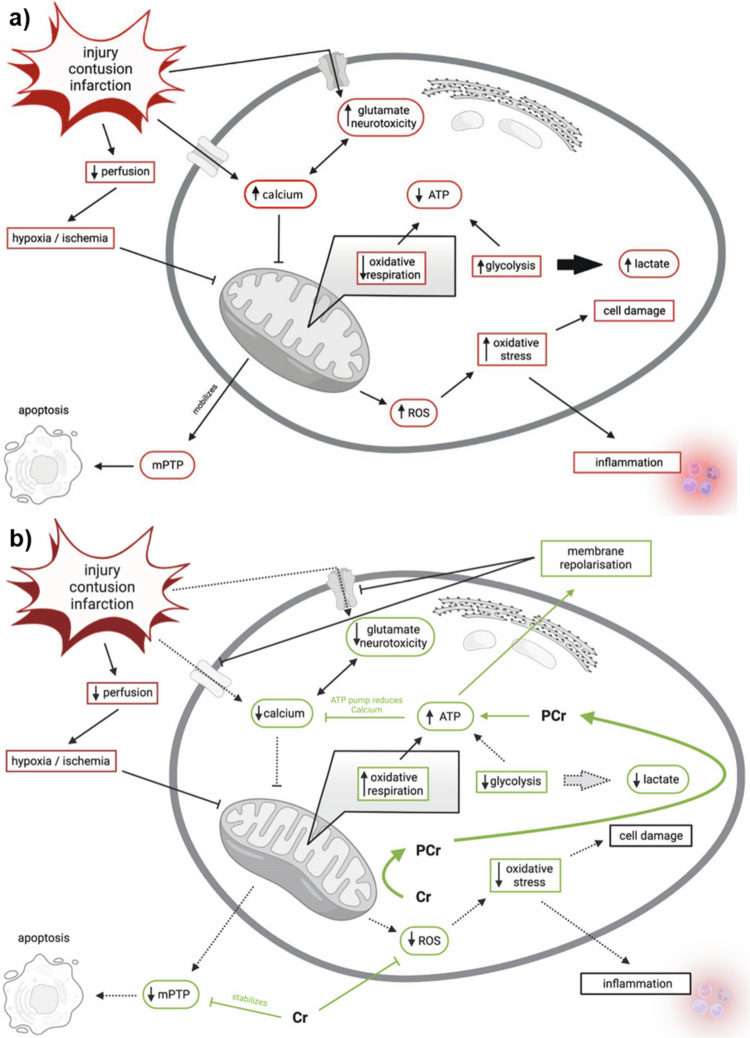
a-b. Cellular events following injury, infarction, or contusion leading to mitochondrial dysfunction: (a) and the modulatory effects of creatine on these processes; (b) Green arrows and boxes indicate stimulation or increases in the creatine/phosphocreatine (Cr/PCr) system, red boxes indicate inhibition or reductions, and dotted lines represent indirect effects of Cr/PCr on cellular pathways (adapted from Ainsley Dean et al. [79] and cited by Marshall et al. [139], pages 4–5).

The sport of soccer and many other sports have ongoing opportunities for players to experience head impacts resulting in concussions and mild traumatic brain injury. A common mechanism for these injuries involves mechanical shear stress on neurons which triggers an intracellular influx of calcium and glutamate [79] and can disrupt mitochondrial energy production. Systemic alternations in energy production, acidifies the cell alongside a fostering of free radical production and inflammation leading to a downstream increase in cell apoptosis via the mitochondrial permeability transition pore (mPTP). Alleviations to this progression may be able to be achieved if the cellular creatine stores are sufficiently filled (Figure 3a,b) due to the cell’s greater potential to maintain short-term energy balance (i.e. buffer function of PCr) and maintain a stable gradient of calcium and glutamate [139].

**Figure 4.**
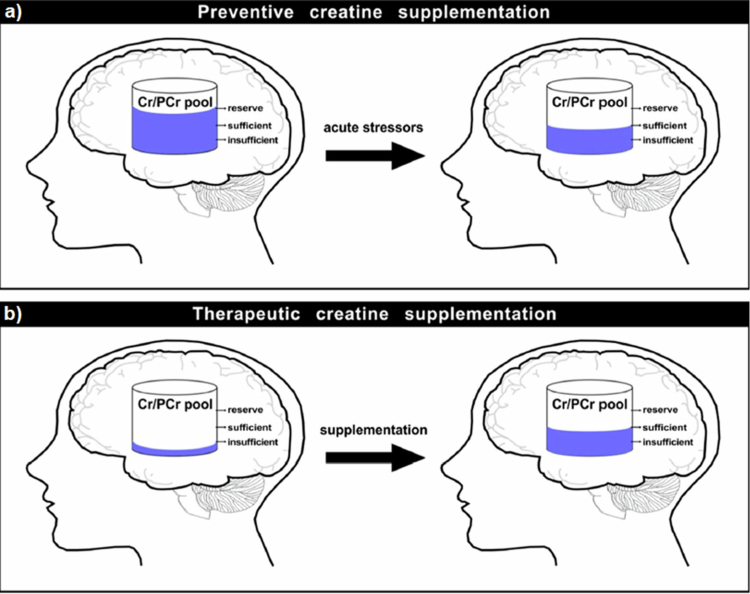
(a–b) Overview of the potential role of creatine supplementation in cognitive function, illustrating preventive effects (a) and therapeutic applications [81].

It remains unclear whether these neuroprotective mechanisms translate to other cell types, in particularly, cardiomyocytes, in humans. To this point, previous experimental studies have demonstrated reduced arrhythmogenic susceptibility following cardiac hypoxia under creatine-augmented conditions [140–145], which provides additional data to highlight the potential for creatine to support vascular and cardiovascular outcomes. Future research more directly in athletic populations should be strongly considered to identify the extent to which a clinically meaningful cardioprotective effects may exist for athletic populations. Finally, and in support of creatine’s potential to support cognitive function during acute stress such as sleep deprivation or hypoxia is the potential efficacy of a prophylactic dose of creatine administered before acute stressors (Figure 4a,b). Early, but limited research has highlighted that attention and reaction speed can be favourably impacted [146,147] while additional experimental approaches must be employed to best understand what impact creatine availability may have on mitigating any detrimental outcomes in stressful environments such as these and others. A study by Cook et al. [148] supplemented elite rugby players with either two doses of caffeine or creatine while complete a sport-specific passing task while sleep deprived. While interest remains very high for the ability of creatine to support brain health and cognitive performance, particularly during the rigours of sport there is still a lack of clarity regarding the most appropriate dosing regimen and form of creatine for tissues such as the brain or for creatine's ability to augment cognition. Importantly, the brain and heart tissue have their own CK isoforms and shuttle systems, which fundamentally alter the absorption kinetics beyond what is firmly established for tissues like skeletal muscle. Due to these key differences, tissue saturation does not occur to the same extent as in muscle. Therefore, one must ask, should one of the goals for creatine supplementation in sporting contexts such as soccer be to prepare the system for bioenergetic stressors, whether they are intense exercise for prolonged periods combined with pervasive cognitive and motor skills demands or the unknown head trauma or to respond to these situations? [81]

Other areas exist where creatine may support the health and performance of many different types of athletes. For example, creatine supplementation has been shown to effectively counteract inflammatory processes in the gut that are associated with chronic inflammatory bowel disease. Creatine also supports the intestinal immune system and may enhance intestinal mucosal integrity by strengthening the enterocytes [149]. Indeed, it is well known that prolonged exposure to high training intensities can cause intestinal leakage [150]. It is plausible that creatine supplementation may support gastrointestinal integrity to help mitigate or counteract the development of increased intestinal permeability (“leaky gut”).

### 6.4. Practical implications for soccer and other team sports

According to current knowledge, soccer athletes do not require loading and maintenance phases, particularly if intended benefits are framed across the entire duration of a soccer season. Instead, a continuous intake of 2–3 g (0.03 g/kg body) of CrM is advisable. At this dosage, full saturation of creatine stores can be expected within 28 days and is sustained if this daily dose persists. In conclusion, creatine provides several benefits for soccer athletes. As an ergogenic aid for training and matches, creatine enhances the recovery capacity for anaerobic performance. It also boosts cognitive and technical skills under stressful conditions. As a protective agent against acute emergencies or insults, creatine helps offset short-term energy deficits, protecting neurons from cell death in cases of mild traumatic brain injury and possibly safeguarding cardiomyocytes from arrhythmogenic events while creatine can also serve as an immunomodulator and anti-inflammatory, counteracting, among other things, sports-related changes in the intestinal mucosa.

## 7. Safety of creatine supplementation

CrM is considered generally recognised as safe (GRAS) by the Food and Drug Administration (FDA) in the United States [151] and is the only form of creatine approved for sale in the United States, Canada, Europe, Australia, Japan, the Republic of Korea, and China [2]. Hundreds of clinical trials have been conducted on CrM in humans, including healthy and medically managed populations, with no clinically significant adverse events reported and few, if any, side effects mentioned [1,2,152,153]. Despite this safety profile, anecdotally reported side effects and myths about creatine supplementation permeate the internet on media platforms, social media, and companies marketing different “forms” of creatine claiming to be more effective with fewer side effects [2,152] despite being discredited [1,2,154–156].

Kreider et al. [157] published the most comprehensive safety analysis to date of 685 clinical trials involving creatine supplementation in humans, which included over 26,000 participants. The methodological scope of this analysis included: (1) evaluating the prevalence of studies reporting side effects for placebo and creatine-supplemented groups; (2) examining the frequency of 35 specific side-effect categories ranging from gastrointestinal issues to neurological symptoms (Kreider et al. [157]); (3) assessing the prevalence of adverse event reports mentioning creatine and the accuracy surrounding those reports; and (4) a social media analysis to assess public perceptions about creatine and side effects.

Nearly all studies (95%) provided CrM at an average dose of 0.17 [0.16, 0.17] g/kg/d (about 12.5 g/d) for 64.7 [52.0, 77.3] days in studies lasting up to 14 years. Side effects were reported in 13% (86/652) of studies in placebo groups and 14% (94/685) of studies in creatine supplemented groups, with no significant differences observed between the groups (*p* = 0.776). There was a slightly higher percentage of studies reporting gastrointestinal (GI)/abdominal issues (Placebo 4.3%, Creatine 4.9%, *p* < 0.001) and muscle cramping/pain (Placebo 0.9%, Creatine 2.9%, *p* = 0.008) with creatine supplementation, but not when the total number of participants in these studies was evaluated. Multivariate analysis of 35 side effects and 14 clinical markers showed no difference (*p* = 0.340) and no difference in the total frequency of side effects reported among participants (placebo 4.2%, creatine 4.6%, *p* = 0.828). These findings support previous reports [45,60,92,93,95,113,114,152,158–175] and the long-term study conducted in Parkinson's patients [176] which reported that creatine supplementation does not increase the frequency of side effects when compared to individuals taking placebos.

This safety analysis also included 28.4 million adverse events reports (AERs) in AER databases in the U.S. [177], Canada [178], Australia [179], Europe [179] as well as the Side Effect Resource (SIDER) [179], which revealed that the mention of creatine in these databases was rare (0.00072%). Specifically, 46.3% of Centre for Food Safety and Applied Nutrition Adverse Events Reporting System (CAERS) reports had no creatine in the products listed, and 63% of AERs with creatine in the product involved the use of other types of creatine or ingestion with other supplements or drugs bringing to light the deeper context often requiring when drawing conclusions from findings in these databases. As such, this report and many others highlight the strong safety profile for CrM use when used within recommended dosages.

## 8. Conclusions

Creatine stands as one of the most rigorously studied and scientifically validated nutritional supplements. Its consistent ability to enhance high-intensity performance, training adaptations, and recovery across a wide range of populations remains among its most consistently supported applications as an ergogenic aid. Beyond its well-established effects on muscle strength, power, and lean mass, creatine use has more recently been investigated in relation to endurance performance, neuroprotection, bone health, and thermoregulation. The growing body of evidence in tactical and occupational populations suggests potential roles for creatine in supporting key performance attributes, cognitive resilience, and overall health in demanding environments, inside and outside of sport. Likewise, research in team-based sports such as soccer continues to indicate possible improvements in sprint capacity, technical performance, and physiological resilience, although the magnitude and consistency of these effects may vary across contexts and study designs, providing further diversification to creatine's potential within athletic populations.

Future research should prioritise mechanistic exploration of creatine's role in brain metabolism, including its influence on cerebral PCr availability, mitochondrial energetics, neurotransmitter regulation, and neuroinflammatory signalling, particularly under conditions of sleep deprivation, hypoxia, or traumatic stress. Greater attention is also warranted to characterise individual response variability, including baseline intramuscular creatine stores, habitual dietary creatine intake, fibre-type distribution, gut absorption kinetics, and potential genetic polymorphisms (e.g. SLC6A8 transporter variants) that may explain “responder” and “non-responder” phenotypes. Additionally, well-powered trials designed to examine sex-specific responses, hormonal influences across the menstrual cycle and menopause, age-related differences in creatine retention and muscle accretion, and the interaction between vegetarian or low-creatine diets and supplementation strategies are needed to refine personalised recommendations.

Across hundreds of clinical trials involving diverse populations, CrM has demonstrated a strong safety profile while consistently supporting improvements in strength, power, lean mass, and high-intensity performance outcomes. Although ongoing surveillance in clinical and aging populations remains important, the convergence of efficacy and safety data positions creatine as a well-supported ergogenic aid whose broader applications for health and resilience continue to evolve with emerging evidence.
